# *BvBZR1* improves parenchyma cell development and sucrose accumulation in sugar beet (*Beta vulgaris* L.) taproot

**DOI:** 10.3389/fpls.2025.1495161

**Published:** 2025-02-03

**Authors:** Ningning Li, Wei Wang, Xiaotong Guo, Yaqing Sun, Guolong Li, Shaoying Zhang

**Affiliations:** ^1^ Sugar Beet Physiological Research Institute, Inner Mongolia Agricultural University, Hohhot, China; ^2^ Scientific Research Center, Shanxi Pharmaceutical Vocational College, Taiyuan, China

**Keywords:** *BvBZR1*, taproot development, transgenic sugar beet, parenchyma cells, sucrose accumulation

## Abstract

BRASSINAZOLE-RESISTANT (BZR) transcription factors, key elements of brassinolide (BR) signal transduction, play an important role in regulating plant growth and development. However, little is known about the molecular regulatory mechanism of BZR in sugar beet taproot growth. In this study, *BvBZR1* expression was significantly induced by exogenous BR treatment. Transgenic sugar beet overexpressing *BvBZR1* exhibited a higher taproot diameter compared with the wild type, mainly due to a significant enhancement in the spacing between cambial rings by increasing the size and layers of parenchyma cells. *BvBZR1* regulated the expression of *BvCESA6*, *BvXTH33*, *BvFAD3*, and *BvCEL1* and enhanced cell wall metabolism to promote sugar beet taproot growth in parenchyma cells and the development of each cambium ring. In addition, *BvBZR1* overexpression significantly increased the accumulation of sucrose and soluble sugars in the taproot, which was attributed to its ability to regulate the expression of *BvSPS* and *BvINV* and improve the activity of BvSPS, BvSS-S, BvSS-C, and BvINV enzymes in each cambium ring and parenchyma cell in the sugar beet taproot. These results suggest that *BvBZR1* can regulate the expression of genes related to cell wall and sucrose metabolism, improve corresponding enzyme activity, and promote the development of each cambium ring and parenchyma cell, thereby promoting the growth and development of sugar beet taproots.

## Introduction

Sugar beet (*Beta vulgaris* L.), a biennial herbaceous plant belonging to the family Chenopodiaceae, is a crucial industrial crop for sugar production and provides approximately 30% of the world’s annual sugar production (Zhang et al., 2017; Li et al., 2022). In the first year of the biennial life cycle of sugar beet, the fleshy taproot develops into 12–15 cambium rings and stores a large amount of sucrose in the concentric rings of vascular tissue originating from the secondary cortex during early root development as well as in parenchyma cells that increase in number and become larger during growth (Rae et al., 2005). Among the cambium rings, the greatest contributions to root development are from rings 1 and 2, whereas rings 3–8 show progressively less activity; rings 1–6 make up approximately 75% of the storage root (Elliott and Weston, 1993). The fleshy taproot, as an important harvesting organ in beet, has always been the focus of traditional breeding for its increased sugar content and yield (Dohm et al., 2014). Therefore, understanding the regulatory mechanisms underlying taproot growth will allow for the engineering of new sugar beet cultivars with high yield and quality.

Brassinolides (BRs), plant-specific hormones, are involved in a wide range of biological processes during plant growth and development, such as root hair formation, lateral root initiation, cell division and elongation, maintenance of meristem size, vascular bundle differentiation, pollen fertility, and gravitropic and stress responses (Wei and Li, 2016; Nolan et al., 2020; Chen et al., 2022). Recently, the mechanism by which BR regulates root growth and development has been confirmed in various plants, including *Phyllostachys pubescens* (Wang T. et al., 2019; Guo et al., 2020), *Arabidopsis thaliana* (Vukasinovic et al., 2021), *Eriobotrya japonica* (Su et al., 2021), *Solanum lycopersicum* (Bai et al., 2021), *Oryza sativa* (Huang et al., 2022), *Triticum aestivum* (Avalbaev et al., 2021; Xu et al., 2022), and *Beta vulgaris* (Wang et al., 2021; Guo et al., 2023). For example, exogenous low concentrations of BR significantly stimulated root elongation in wild-type (WT) *Arabidopsis*, and this root growth promotion effect was more pronounced in BR-deficient *dwf1-6* mutants (Müssig et al., 2003). Exogenous spraying of BR promotes the development of parenchyma cells and the secondary xylem, thereby increasing the diameter of the sugar beet taproot and regulating the expression of cell wall biosynthesis-related genes *BvXTH33*, *BvSHV3*, *BvCESA6*, *BvPARVUS*, and *BvCEL1* (Wang et al., 2021). In addition, the increase in endogenous BR due to the overexpression of *BvCPD* significantly improved cell wall components and promoted the development of parenchyma cells and vascular bundles, thereby playing an important role in promoting sugar beet taproot growth and development (Guo et al., 2023). These results indicate that both endogenous and exogenous BR can significantly improve the development of parenchyma cells and the secondary xylem, thereby promoting sugar beet taproot growth and development. However, little is known about how BR regulates the development of sugar beet taproot parenchyma cells and secondary xylem. Therefore, research on the key elements of BR signal transduction and its role in modulating root growth and development can provide insight into the molecular mechanism of taproot growth and development and will also provide a theoretical basis for cultivating high-yield and high-quality sugar beet varieties.

Recently, the BR signal transduction pathway has been extensively investigated. BRs bind to BRASSINOSTEROID-INSENSITIVE 1 (BRI1), an extracellular leucine-rich repeat (LRR) domain of cell transmembrane receptor kinase, which enhances BRI1 heteromerization with BRI1-associated kinase 1 (BAK1) (Wang et al., 2001; Sun et al., 2013) and then activates BR SIGNALING KINASE 1 (BSK1) and CONSTITUTIVE DIFFERENTIAL GROWTH 1 (CDG1) (Tang et al., 2008; Kim et al., 2011). Activated BSK1 and CDG1 further regulate BRI-SUPPRESSOR 1 (BSU1) and act on BRASSINOSTEROID-INSENSITIVE 2 (BIN2), leading to the activation of transcription factors BRASSINAOLE-RESISTANT 1 (BZR1) and BRIEMS-SUPPRESSOR 1 (BES1) by PROTEIN PHOSPHATASE 2A (PP2A) (Yu et al., 2008; Kim et al., 2009; Tang et al., 2011). BZR1 recruits the BRAHMA-associated SWI/SNF (BAS) complex to open chromatin and mediate BR-induced transcriptional activation of growth-promoting genes (Zhu et al., 2024). BZR1, a bHLH transcription factor, plays an important role in regulating plant growth and development as well as stress responses (Oh et al., 2012; Kono and Yin, 2020; Nolan et al., 2020, 2023). For instance, BZR1 can regulate plant hypocotyl elongation under the treatment of light, temperature, auxin, gibberellin, and sugar (Bai et al., 2012; Oh et al., 2012, 2014). BZR1 is also involved in regulating plant fertility development, playing an important role in regulating anther and seed development (Ye et al., 2010; Jiang and Lin, 2013). The expression activity of BZR1 at the root tip can significantly regulate the differentiation of root meristem cells and participate in the regulation of xylem differentiation, cell division, and elongation (Chaiwanon and Wang, 2015; Wei and Li, 2016; Nolan et al., 2023). In addition, BZR1 participates in various stress responses and plays an important role in responding to stress, such as heat, cold, drought, and pathogen infection (Fan et al., 2014; Chen et al., 2017; Ramirez and Poppenberger, 2020; Nolan et al., 2020). Although it is known that BZR1 can regulate various growth and development processes in plants, the underlying mechanism by which BZR1 regulates sugar beet taproot parenchyma cells and secondary xylem development is still poorly understood. Therefore, elucidating the molecular mechanism of BvBZR1 in regulating the growth and development of sugar beet taproot will provide a new perspective for molecular genetic breeding of high-yield and quality sugar beets.

In our previous transcriptome analysis, significant differential expression of BvBZR family members was observed during the growth and development of the sugar beet taproot, suggesting that these family members may play an important role in taproot enlargement (Zhang et al., 2017). Subsequently, we performed a genome-wide investigation of *BvBZR* genes in sugar beet. Six *BvBZR* gene family members were identified in the sugar beet genome, clustered into 3 subgroups and distributed on 4 chromosomes. The *Bv1_fxre* gene is located on chromosome 1 and encodes the BvBZR1 protein localized in the nuclei. The *BvBZR1* gene is mainly expressed in the leaves and taproot and has the highest expression during the period with the maximum growth rate of the taproot (Wang W. et al., 2019). In the present work, we constructed transgenic sugar beet overexpressing *BvBZR1* using *Agrobacterium*-mediated genetic transformation of cotyledon nodes and elucidated the function of *BvBZR1* in the growth and development and sucrose accumulation of sugar beet taproots through morphological, anatomical, and molecular biological studies. Preliminary exploration of the molecular regulatory mechanism of *BvBZR1* regulating taproot growth and development was conducted through RNA-sequencing (RNA-seq) and yeast single hybrid technology. This study provides insight into the molecular mechanism of *BvBZR1* regulation of sugar beet taproot growth and development and sucrose accumulation and provides a candidate gene for the genetic improvement of high yield and quality in sugar beet.

## Materials and methods

### Plant materials

Sugar beet cultivar ‘BS02’, a homozygous cultivar with a high sucrose content, was bred by the Sugar Beet Physiological Research Institute, Inner Mongolia Agricultural University, China, and used in this study. The seeds were sown in vermiculite with one seedling per 8×8×8 cm^3^ float tray, and the seedlings were grown in an artificial climate chamber at 22°C with 60% humidity and a 16/8 h light/dark cycle. Plants with eight leaves were treated with 0.1 mg/L of epibrassinolide (Sigma, USA) or 20 µmol/L of Brz (Sigma, USA) for 4 h. The control group was treated with water. The samples were collected to investigate *BvBZR1* gene expression patterns.

Sterile BS02 seedlings were obtained through the following steps: the seeds were artificially sanded to ensure a smooth seed shell. Seeds were washed for 30 min, treated with 0.1% HgCl_2_ solution for 10 min, rinsed five times with sterile water, dried on a filter paper for 5–10 min, and planted in germination medium (1 mg/L 6-BA + 8.0 g/L agar; pH 5.8). Seeds were cultured at 22°C with a 16 h light/8 h dark photoperiod and a photon flux density of 45 mmol m^−2^ s^−1^. Ten-day-old sugar beet seedlings were used for genetic transformation experiments.

### Genetic transformation of sugar beet

The *BvBZR1* gene (*Bv1_fxre*) was isolated in our previous research (Wang W. et al., 2019). To analyze the function of *BvBZR1*, a pCAMBIA1300-BvBZR1 vector was constructed. The open reading frame (ORF) of *BvBZR1* was amplified using the BvBZR1-BamHI-F and BvBZR1-SalI-R primers (**Supplementary Table S1**) and inserted between the *Bam*HI and *Sal*I sites of the pCAMBIA1300 vector under control of the CaMV 35S promoter to generate pCAMBIA1300-BvBZR1. pCAMBIA1300-BvBZR1 was introduced into *Agrobacterium tumefaciens* (strain LBA4404) using the freeze–thaw method (Jyothishwaran et al., 2007), and sugar beet was transformed using the cotyledon node infiltration method described by Guo et al. (2023). Briefly, 10-day-old sterile seedling cotyledon nodes were cut off and pre-cultured (MS + 30 g/L sucrose + 1 mg/L 6-BA + 0.2 g/L casein + 7 g/L agar) for 24 h, followed by 10 min of *Agrobacterium* infection, and incubated on co-culture medium (MS + 30 g/L sucrose + 1 mg/L 6-BA + 0.2 g/L casein + 100 µM AS + 7 g/L agar) for 2–3 days in the dark. After washing the cotyledonary nodes, they were cultured on a suppressive medium (MS + 30 g/L sucrose + 1 mg/L 6-BA + 0.25 mg/L NAA + 0.5 mL/L PPM + 8.0 g/L agar + 400 mg/L temetin) for 5–7 days and then transferred to a screening medium (MS + 30 g/L sucrose + 1 mg/L 6-BA + 0.25 mg/L NAA + 0.5 mL/L PPM + 8.0 g/L agar + 6 mg/L hygromycin) for 15–20 days to screen resistant beet shoots. The resistant shoots were then transferred to a rooting medium (MS + 30 g/L sucrose + 2 mg/L NAA + 0.5 mL/L PPM + 8.0 g/L agar) to induce root formation. After 1 month, the seedlings were refined and transplanted into nutrient soil. Transgenic lines were confirmed using genomic PCR. The vector-specific primers Hgy2-F and Hgy2-R (**Supplementary Table S1**) were used for PCR under the following conditions: denaturation at 94°C for 10 min, 33 cycles of 94°C for 1 min, 55°C for 30 s, and 72°C for 1 min, followed by a final extension period at 72°C for 5 min. Selected hygromycin-resistant plants were used for further validation and subsequent functional identification.

### Real-time PCR

To quantify *BvBZR1* expression in sugar beet under BR treatment and transgenic plants, real-time PCR was performed using *BvBZR1*-specific primer sets (BvBZR1-RT-F/R; **Supplementary Table S1**) with the *BvActin* gene as an internal control. To examine the expression of genes related to cell wall synthesis and sucrose metabolism in transgenic sugar beet, cDNA was synthesized using RNA extracted from different tissues from WT and transgenic plants. The expression levels of the *BvCEL1*, *BvCESA6*, *BvFAD3*, *BvXTH33*, *BvSPS*, and *BvINV* genes were quantified using real-time RT-PCR. The *BvActin* gene was used as an internal control. The sequences of all primers used are listed in **Supplementary Table S1**. Total RNA was reverse-transcribed into first-strand cDNA using the TransScript One-Step gDNA Removal and cDNA Synthesis Super Mix (TransGen Biotech, Inc.) according to the manufacturer’s protocol. Three separate cDNA sample dilutions were used as templates for real-time PCR analysis. The cDNA was amplified using TransStart Tip Green qPCR SuperMix (TransGen Biotech, Inc.) in a 480 II real-time PCR Light Cycler (Roche) under the following reaction conditions: denaturation at 95°C for 30 s, followed by 48 cycles of 95°C for 10 s, 58°C for 15 s, and 72°C for 20 s. The threshold cycle (CT) values of triplicate samples were averaged, and fold-change values in the transcription level of the target gene relative to the reference gene were calculated using the comparative CT (2^–△△CT^) method, as previously described by Livak and Schmittgen (2001). All experiments were repeated three times using cDNA prepared from three batches of plants.

### RT-PCR analysis

To investigate *BvBZR1* expression in transgenic sugar beet plants, RT-PCR was performed using the specific primers BvBZR1-RT-F and BvBZR1-RT-R (**Supplementary Table S1**). The *BvActin* gene was used as an internal control. RNA was prepared from leaves of transgenic sugar beet seedling according to the operating instructions of the TransZol Up Plus RNA Kit (TransGen Biotech, Inc.). The cDNA was synthesized from 1 µg of total RNA at 42°C for 30 min with 1 µL of oligo (dT) using an RT-PCR kit (TransGen Biotech, Inc.). The PCR mix included 2.5 µL of 10×TransStart Taq buffer, 2 µL of 2.5 mM dNTPs, 2.5 U of TransStart Taq DNA polymerase, 1 µL of 10 mM primers, and 1 µL of cDNA for a 25 µL volume. The reaction conditions were 35 cycles of 94°C for 30 s, 56°C for 30 s, and 72°C for 1 min, followed by a final extension period of 10 min at 72°C.

### Phenotypic determination of transgenic sugar beet

Morphological indicators of sugar beet were measured when transplanted transgenic sugar beet seedlings were incubated in an artificial climate chamber for 80 days. Three plants were sampled from each line. The fresh weight of the total plant, leaf, petiole, and root was determined using an electronic balance. The plant height, leaf length, and root length were measured with a tape measure, and the root diameter was measured with a vernier caliper.

### Histological observations

Samples were collected from the root 1 cm from the root apex. All specimens were fixed in 70% formaldehyde acetic acid for 24 h and dehydrated in an increasing ethanol series. Transverse 12-µm-thick sections were obtained with a rotating microtome, stained with Safranin and Astra Blue, and mounted on paraffin (Maia et al., 2018). The slides were observed under a microscope (Pannoramic DESK, Hungary) and photomicrographed. The images were analyzed with CaseViewer. The following histological parameters were evaluated: ring spacing, area of parenchyma cells, and number of parenchyma cell layers. The distance between the outermost xylem of two vascular forming layers was defined as the spacing between the rings of forming layers. CaseViewer was used to randomly select 10 adjacent cells to determine the total area, and the average area was calculated. The number of parenchyma cell layers between the rings of two forming layers was counted, and ten groups of data were randomly selected for analysis. All experiments had 10 biological replicates.

### RNA-seq for transgenic sugar beet

To investigate the function of *BvBZR1*, total RNA was isolated from 60-day-old WT and transgenic line 2 (OE2) using a total RNA Isolation System (Takara, Shanghai, China) according to the manufacturer’s instructions. The concentration and quality of RNA were determined on a 2100 Bioanalyzer (Agilent Technologies), and 3 mg of RNA from each sample was used for library construction. Sequencing of three independent biological replicates per sample type was performed using the NovaSeq 6000 platform (Illumina). Approximately 4.0 Gb of clean data were generated per sample. Clean reads were mapped to a genome reference (RefBeet-1.1) using BWA (Burrows-Wheeler Alignment Tool) and Bowtie (Dohm et al., 2014). The differentially expressed genes (DEGs) were filtered using the following thresholds: |log^2^(Fold change)| > 1 and adjusted P (q-value) < 0.05. Functional annotation of the DEGs was conducted using the Blast2GO program and the non-redundant protein database (NR; NCBI). A significance level of P ≤ 0.05 was used to confirm Gene Ontology (GO), Kyoto Encyclopedia of Genes and Genomes (KEGG) pathway, and MapMan analysis results.

### Yeast single hybridization

To explore the binding of *BvBZR1* to the promoters of the target genes, a yeast one-hybrid kit was used in accordance with the manufacturer’s protocol (Clontech Laboratories, Mountain View, CA, USA). The promoters of the sugar beet genes *BvXTH33* and *BvCESA6* were cloned into the pAbAi vector to acquire pAbAi-bait vectors. These recombinant pAbAi-bait and pAbAi-53 vectors were transformed into Y1HGold yeast, and the background aureobasidin A (AbA) expression of the bait strain was tested. The pGADT7 prey vector harboring the *BvBZR1* ORF (pGAD-*BvBZR1*) was transformed into the bait strain. Y1HGold yeast cells were used as a negative control, and yeast cells co-transformed with pAbAi-53 and pGADT7-p53 vectors were used as positive controls. The transformed yeast cells were grown in a leucine- and uracil-deficient SD medium containing AbA. After culturing at 30°C for 4 days, the plates were photographed.

### Double luciferase assay

The promoters of the sugar beet *BvXTH33* and *BvCESA6* were inserted into the pGreenII0800-LUC vector as reporter plasmids, and the pGreenII62sk-BvBZR1 were used as effectors plasmid in dual-LUC assays. The mixed effector and reporter plasmids were electrotransformed into Agrobacterium GV3101, and injected into the lower epidermis of tobacco leaves. After three days of low light culture, the leaves were collected and the cells were lysed. The activity of LUC/REN was assessed by employing a kit for dual-luciferase reporter assay (E1910, Promega, Madison, WI, USA). Each sample was measured in triplicate.

### Physiological phenotype determination

Sixty days after sugar beet transplantation, samples were collected from the roots, stems, leaves, and vascular rings and inter-rings of one to three layers of the taproot. The contents of sugar, total soluble sugar, and reducing sugar were measured according to the manufacturer’s instructions (Jiangsu Addison Biotechnology Co., China). The activities of BvCEL, BvCESA, BvFAD, BvXTH, BvSPS, BvSS-C, BvSS-S, and BINV were measured using corresponding assay kits (all from the Jiancheng Bioengineering Institute, Nanjing, China) according to the manufacturer’s instructions.

### Statistical analysis

Data are expressed as means ± standard deviations. Statistical differences were assessed by one-way analysis of variance (ANOVA) using Duncan’s multiple range test (MRT). The significance of differences between the means was assessed using a probability (P) value of < 0.05. All analyses were performed using SPSS 18.0 software (ver. 19; SPSS, Inc., Chicago, IL, USA).

## Results

### *BvBZR1* was significantly induced by BR

**Figure 1:**
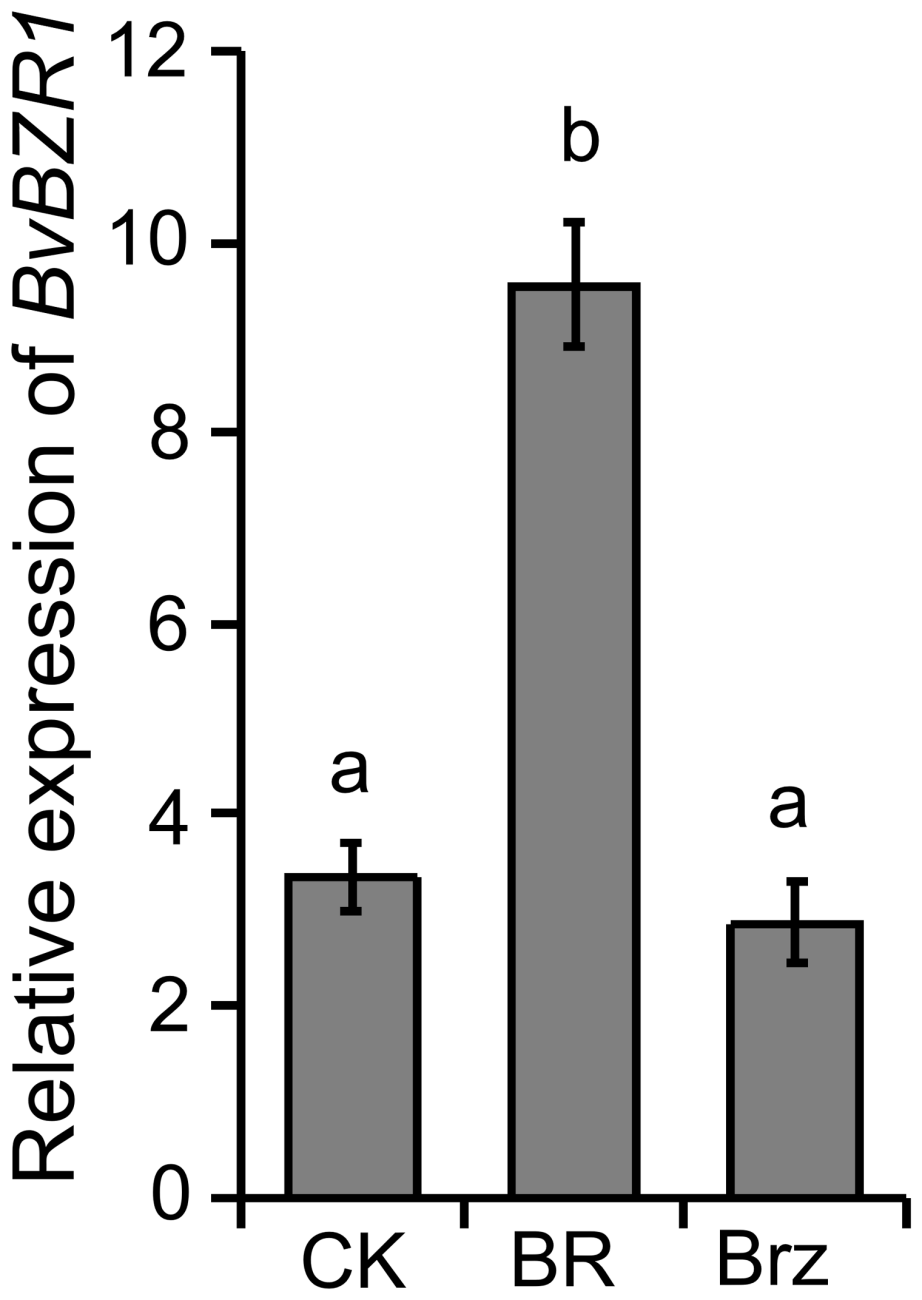
The expression level of *BvBZR1* after BR treatment. Roots were sprayed with BR, Brz, or water (CK) for 4 h to determine whether *BvBZR1* expression was regulated by exogenous BR in sugar beet. Values not connected by the same lowercase letter are significantly different (Duncan’s multiple range test, P < 0. 05).

Our previous research showed that BRs increased the spacing between the cambial rings by increasing the size of parenchyma cells between the rings and ultimately increasing sugar beet taproot diameter, and some cell wall biosynthesis-related genes were significantly induced by BR (Wang et al., 2021). To determine whether the expression level of *BvBZR1* was regulated by exogenous BR in sugar beet, the roots were sprayed with BR, Brz, or water for 4 h. As shown in **Figure 1**, compared with the control group, the expression level of *BvBZR1* was significantly increased after spraying with BR, whereas it slightly decreased after Brz treatment, suggesting that BR could promote BvBZR1 expression.

### *BvBZR1* in transgenic sugar beet

**Figure 2:**
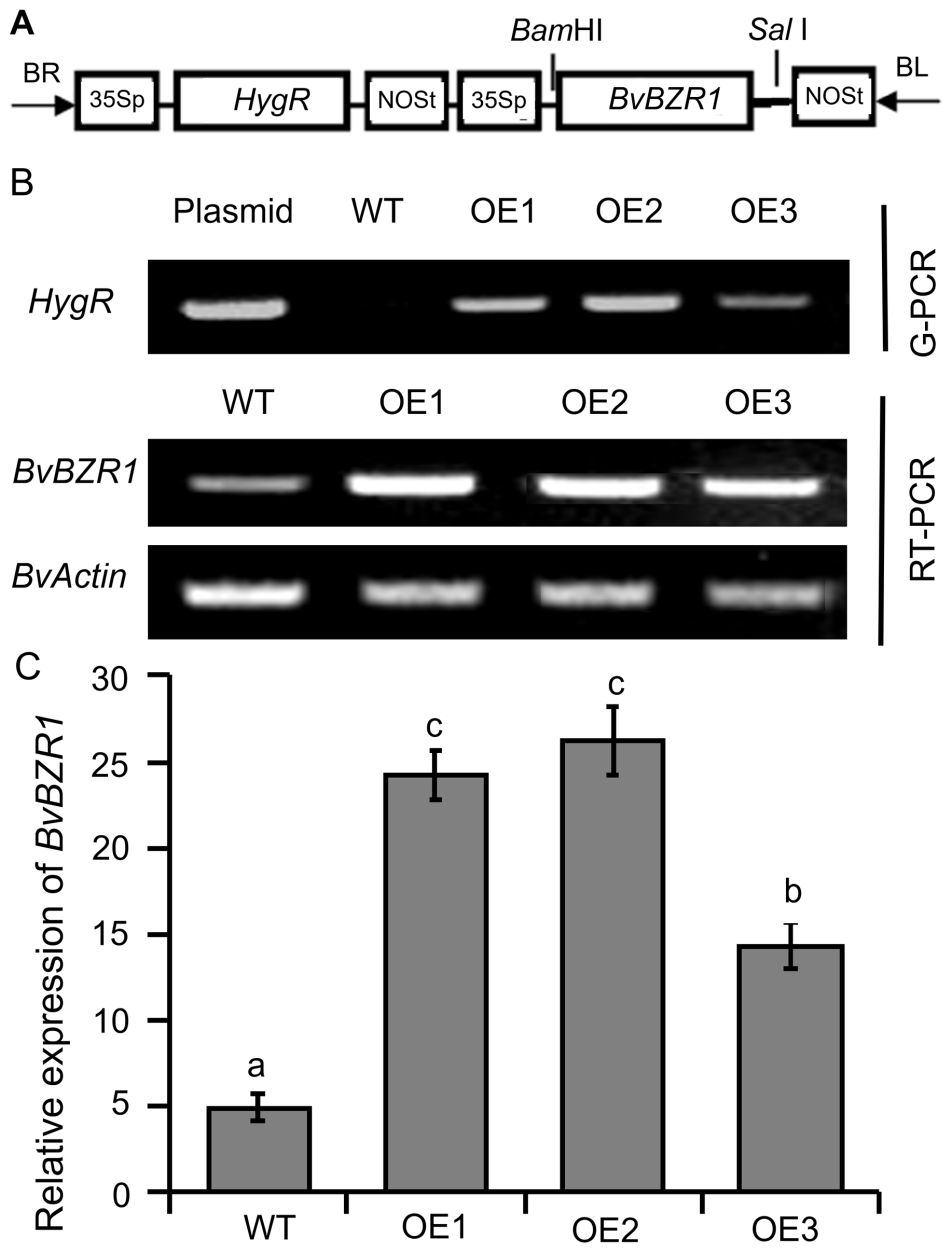
Molecular identification of BvBZR1-transformed sugar beet lines. **(A)** T-DNA region of the vector pCAMBIA1300-BvBZR1 used to produce transgenic *BvBZR1* plants. 35Sp, cauliflower mosaic virus 35S promoter; NOSp, nopaline synthase promoter; NOSt, nopaline synthase terminator; HygR, hygromycin gene; BR, right border; BL, left border. **(B)** Genomic-PCR and RT-PCR analyses used to confirm the insertion of *BvBZR1* in the genome and *BvBZR1* expression, respectively. The *BvActin* gene was used as an internal control. **(C)** Real-time RT-PCR analyses used to further precisely quantify the expression levels of *BvBZR1* in leaves of transgenic plants. The *BvActin* gene was used as an internal control. Values not connected by the same lowercase letter are significantly different (Duncan’s multiple range test, P < 0. 05).

To analyze the function of *BvBZR1*, the pCAMBIA1300-BvBZR1 plasmid containing the *BvBZR1* cDNA sequence under the control of the CaMV35S promoter was constructed and introduced into sugar beet by *Agrobacterium-*mediated cotyledon node transformation (**Figure 2A**). After screening for hygromycin resistance, three lines overexpressing *BvBZR1* (OE1, OE2, and OE3) with hygromycin resistance were validated by Genomic-PCR and RT-PCR (**Figure 2B**). Real-time RT-PCR analysis showed that the *BvBZR1* expression levels of transgenic sugar beets were significantly higher than those of the WT (**Figure 2C**).

### *BvBZR1* overexpression promoted plant development and increased biomass in sugar beet

**Figure 3:**
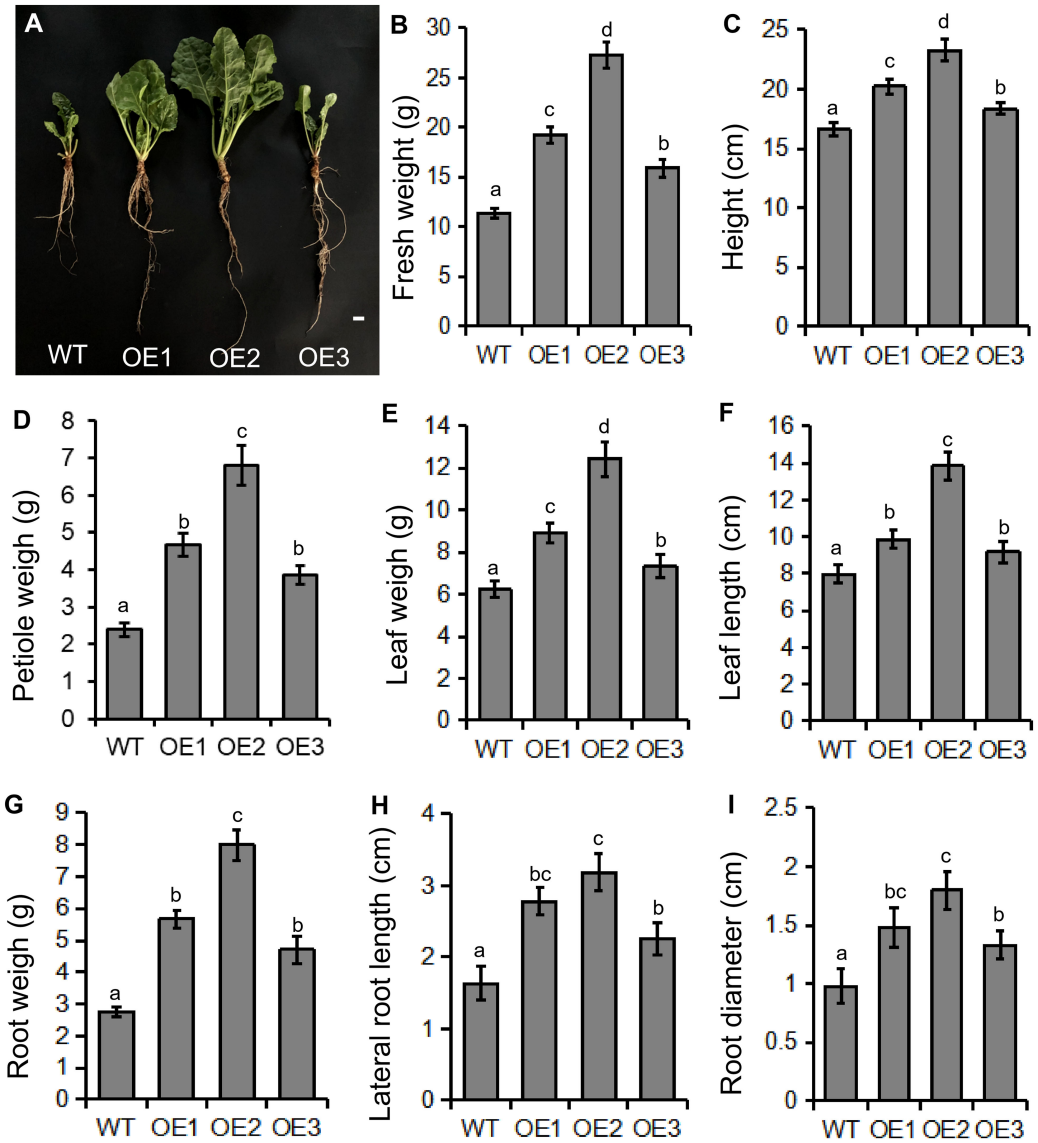
Phenotype determination of transgenic and wild-type (WT) sugar beet. **(A)** Phenotype of 60-day-old transgenic sugar beet overexpressing *BvBZR1* and the WT. Scale bars = 1 cm. The total fresh weight **(B)**, height **(C)**, petiole weight **(D)**, leaf weight **(E)**, leaf length **(F)**, root weight **(G)**, root length **(H)**, and root diameter **(I)** of 60-day-old transgenic sugar beet overexpressing *BvBZR1* and the WT. Data are expressed as means ± standard errors (n = 5). Values with the same letter are not significantly different (Duncan’s multiple range test, P < 0. 05).

Transgenic sugar beet overexpressing *BvBZR1* displayed faster growth and development compared with the WT (**Figure 3A**). The total fresh weight and height of transgenic sugar beet overexpressing *BvBZR1* were significantly higher than those of the WT (**Figures 3B, C**). In the shoot morphology, the petiole weight, leaf weight, and leaf length of transgenic plants were significantly higher than those of WT plants (**Figures 3D–F**). *BvBZR1* overexpression enhanced the root growth of transgenic sugar beet and significantly increased the root weight, root length, and root diameter (**Figures 3G–I**). These results suggest that *BvBZR1* overexpression can promote the root growth of sugar beet by increasing the root length and diameter.

### *BvBZR1* overexpression increased the spacing between cambial rings, layers, and area of the parenchyma cells

**Figure 4:**
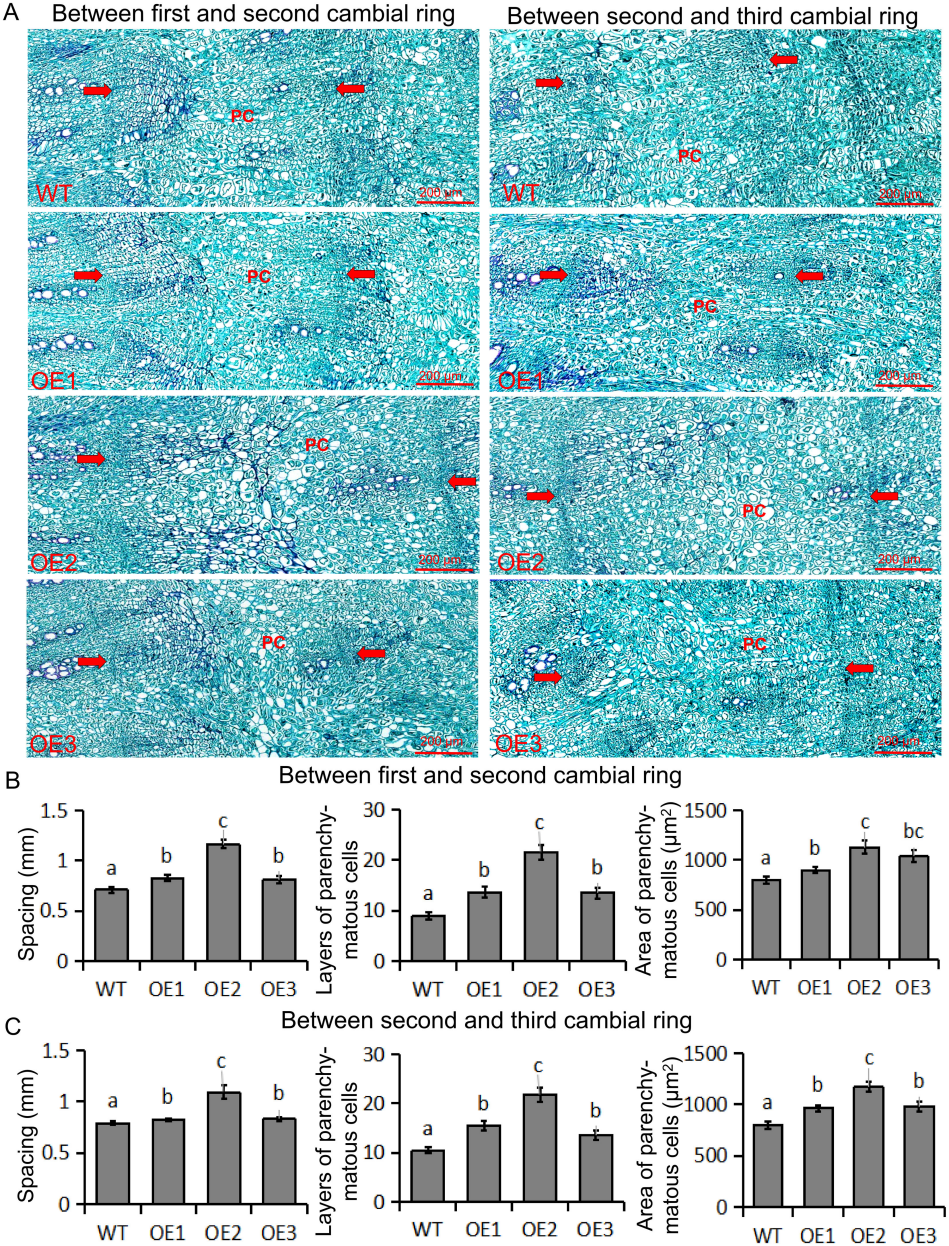
Determination of ring spacing, parenchyma cell size, and cell layer number in the first three rings. **(A)** Cross-section of wild-type (WT) and transgenic sugar beet taproot in the first three ring-forming layers. The distance between the two arrows indicates the cambial rings; the PC indicates parenchyma cells. Scale bar = 200 μm. **(B)** Spacing, area of parenchyma cells, and layers of parenchyma cells between the first and second cambial rings. **(C)** Spacing, area of parenchyma cells, and layers of parenchyma cells between the second and third cambial rings. Data are expressed as means ± standard errors (n = 10). Values with the same letter are not significantly different (Duncan’s multiple range test, P < 0. 05).

To determine how *BvBZR1* promotes the increase in taproot diameter of transgenic sugar beet, we examined taproot cross-sections from transgenic and WT plants (**Figure 4A**). Three cambial rings were found in all groups. The ring spacing of 1st and 2nd formation layers of the taproot in transgenic sugar beet was significantly higher than that in the WT (**Figure 4B**). Compared with the WT, the layers of parenchymal cells of 1st and 2nd cambial rings were significantly increased in *BvBZR1*-overexpressing beet taproots (**Figure 4B**). The parenchymal cells between 1st and 2nd cambial rings of transgenic plants were significantly larger than those of WT plants (**Figure 4B**). Similar results were found for 2nd and 3rd cambium rings (**Figure 4C**). These results suggest that *BvBZR1* overexpression can enhance the spacing between cambial rings by increasing the size and layers of parenchyma cells, thereby increasing the taproot diameter of sugar beet.

### *BvBZR1* regulated the expression of *BvXTH33* and *BvCESA6* genes

**Figure 5:**
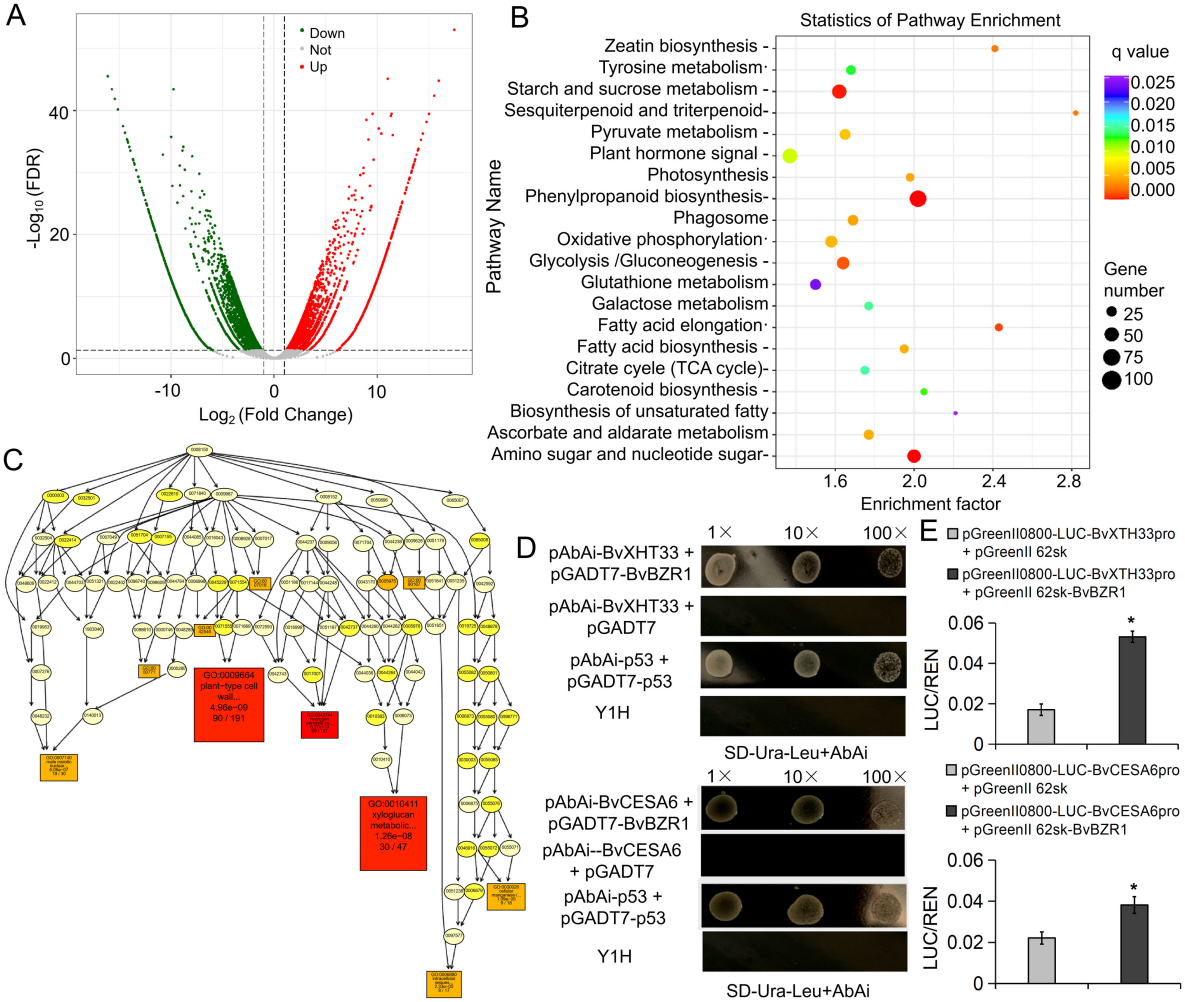
RNA-seq for screening downstream target genes of *BvBZR1* and validation by yeast single hybridization. **(A)** Volcano plot of differentially expressed genes (DEGs). **(B)** Kyoto Encyclopedia of Genes and Genomes (KEGG) enrichment scatter diagram of the DEGs. **(C)** Directed acyclic graph analysis of significantly enriched GO entries in biological processes. Yeast single hybrid **(D)** and dual-LUC assays **(E)** validation of *BvBZR1* binding to the promoter regions of the *BvXTH* and *BvCESA* genes. An asterisk (*) indicates a significant difference (Duncan’s multiple range test, P < 0. 05).

To investigate the function of *BvBZR1*, we performed RNA-seq on WT and OE2, each with three biological replicates. The DEGs were screened according to the |log2 ratio| ≥ 1 (P < 0.001) and false discovery rate ≤ 0.001. We identified 5172 upregulated and 6208 downregulated genes in OE2 versus WT (**Figure 5A**). KEGG pathway analysis was performed using a P-value of less than 0.05 as the cutoff, and the DEGs were significantly enriched in metabolic pathways such as starch and sucrose metabolism, phenylpropanoid biosynthesis, and amino sugar and nucleotide sugar metabolism (**Figure 5B**). Directed acyclic graph analysis was performed on significantly enriched GO entries in cellular components, molecular functions, and biological processes. The results showed that the two largest subcategories were found in the “cellular component” category, including “cell wall” and “plant-type cell wall.” In the “biological processes” category, “plant-type cell wall organization” and “xyloglucan metabolic process” were the most abundant GO terms (**Figure 5C**). In the “molecular function” category, the largest subcategory was “xyloglucan: xyloglucosyl transferase activity.” These results indicate that the *BvBZR1* gene may be involved in regulating plant cell wall tissue metabolism and the xyloglucan metabolic process.

To determine whether *BvBZR1* could directly regulate the expression of genes related to cell wall metabolism, we selected significantly upregulated genes *BvCESA6* and *BvXTH33* from the significantly enriched “plant-type cell wall organization” and “xyloglucan metabolic process” entries for yeast single hybridization validation. *BvBZR1* directly bound the promoter region of the *BvXTH33* and *BvCESA6* genes (**Figure 5D**). The dual-LUC assays further confirmed that the *BvBZR1* has the ability to activate the transcription of *BvCESA6* and *BvXTH33* in tobacco protoplasts (**Figure 5E**).

### *BvBZR1* regulated the expression of genes and the activity of enzymes related to cell wall synthesis to promote cell wall metabolism

**Figure 6:**
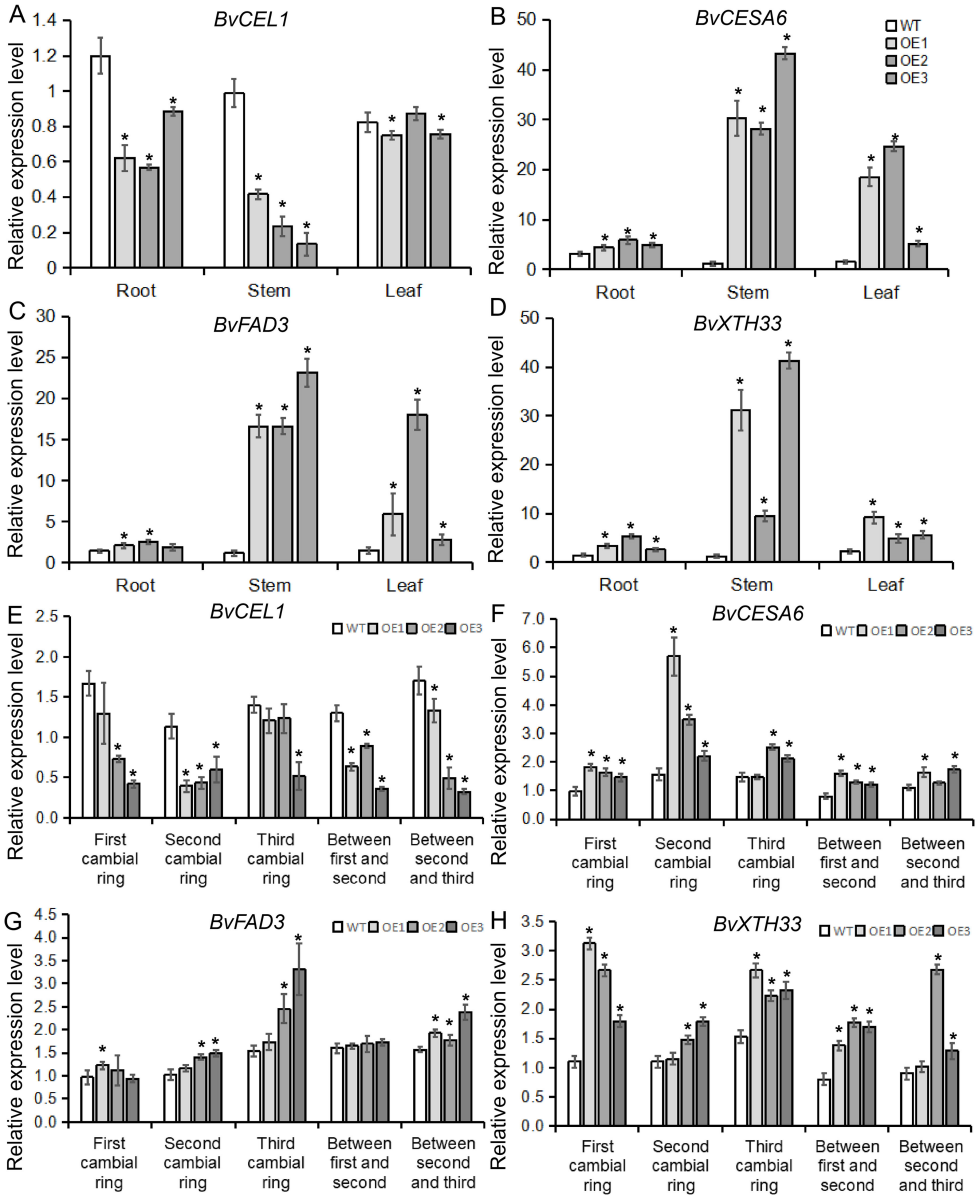
The expression patterns of genes related to cell wall synthesis in BvBZR1 transgenic sugar beet. The expression levels of *BvCEL1* **(A)**, *BvCESA6* **(B)**, *BvFAD3* **(C)**, and *BvXTH33* **(D)** in the roots, stems, and leaves of transgenic plants, and the expression levels of *BvCEL1* **(E)**, *BvCESA6* **(F)**, *BvFAD3* **(G)**, and *BvXTH33* **(H)** in the cambial rings of transgenic plant taproots. Data are expressed as means ± standard errors (n = 3). An asterisk (*) indicates a significant difference (Duncan’s multiple range test: WT vs. transgenic plants, P < 0.05).

**Figure 7:**
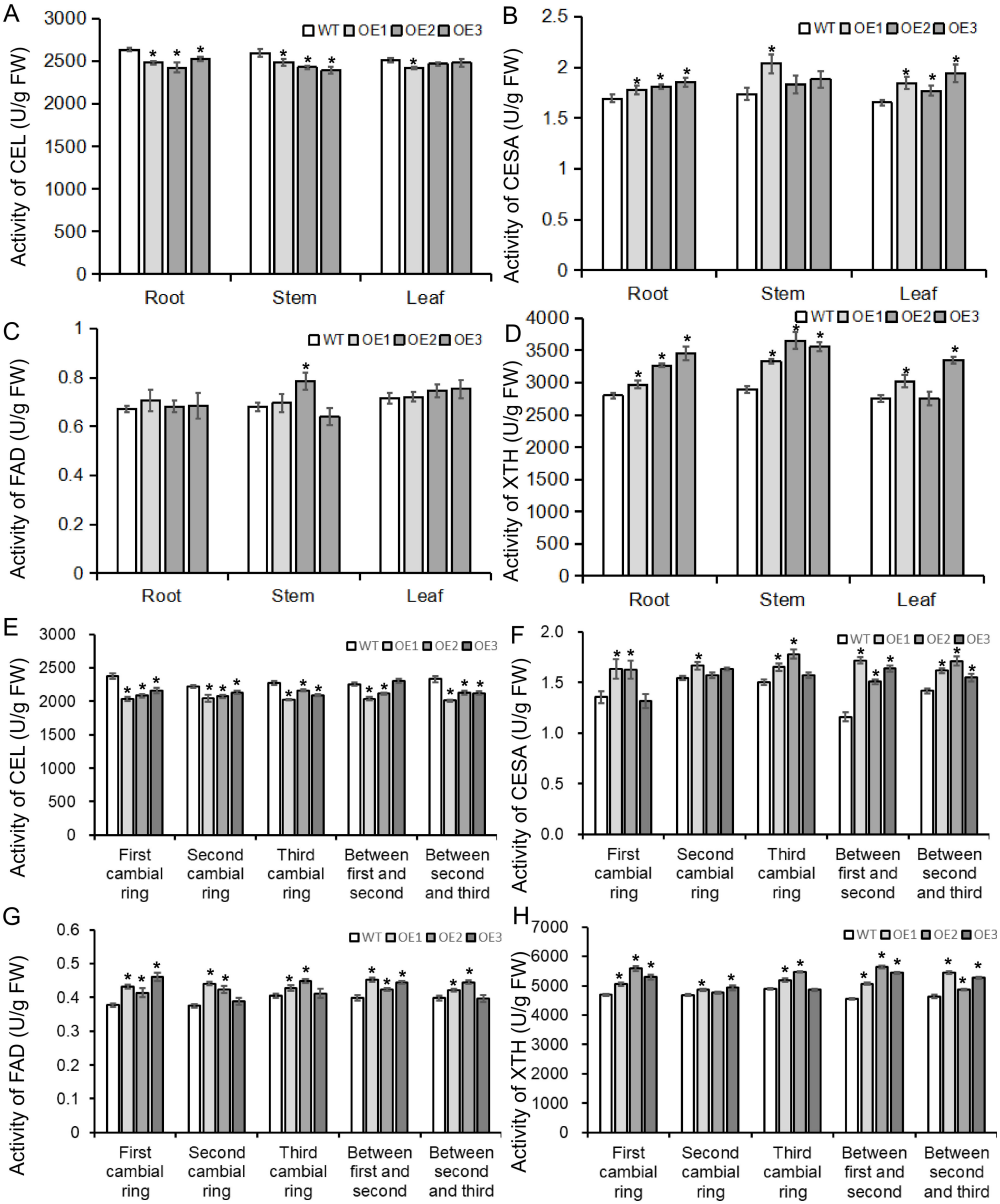
The activity of enzymes related to cell wall synthesis in BvBZR1 transgenic sugar beet. The activities of BvCEL1 **(A)**, BvCESA6 **(B)**, BvFAD3 **(C)**, and BvXTH33 **(D)** in the roots, stems, and leaves of transgenic plants, and the activity of BvCEL1 **(E)**, BvCESA6 **(F)**, BvFAD3 **(G)**, and BvXTH33 **(H)** in cambial rings of transgenic plant taproots. Data are expressed as means ± standard errors (n = 3). An asterisk (*) indicates a significant difference (Duncan’s multiple range test: WT vs. transgenic plants, P < 0.05).

To determine whether the *BvBZR1* gene played a regulatory role in cell wall synthesis, the expression of cell wall synthesis-related genes, including *BvCEL1, BvCESA6, BvFAD3*, and *BvXTH33*, was detected in transgenic sugar beet taproots, stems, and leaves (**Figures 6A–D**). The expression of *BvCESA6, BvFAD3*, and *BvXTH33* in the leaves of transgenic plants was significantly higher than that of the WT. In the stems, the expression of the four genes in transgenic plants was significantly higher than that in the WT. In the roots, overexpression of the *BvBZR1* gene significantly increased the expression levels of *BvCESA6* and *BvXTH33* genes while decreasing the expression levels of *BvCEL1* (**Figures 6A–D**). Similarly, BvCEL, BvCESA, BvFAD, and BvXTH enzyme activities were detected in the roots, stems, and leaves of transgenic plants (**Figures 7A–D**). In the leaves, the BvCESA activity of transgenic plants was significantly increased compared with the WT. In the stems, the BvCEL and BvXTH activities of transgenic plants were significantly higher than those of the WT. In the roots, *BvBZR1* overexpression significantly increased the activity of BvCESA and BvXTH but decreased the activity of BvCEL (**Figures 7A–D**). These results indicate that *BvBZR1* can regulate the expression of the *BvCEL1, BvCESA6*, and *BvXTH33* genes, improve their enzyme activity, and promote taproot cell wall biosynthesis in sugar beet.

To further determine how *BvBZR1* promotes cell wall biosynthesis in the sugar beet taproot, the expression of *BvCEL1, BvCESA6*, *BvFAD3*, and *BvXTH33* was detected in each cambial ring and in parenchyma cells between each cambium ring of transgenic plant taproots. The expression level of the *BvCEL1* gene in parenchyma cells between the first and second/second and third cambium rings and in the second cambium ring of transgenic sugar beet taproot was significantly lower than that of the WT (**Figure 6E**). The expression of the *BvCESA6* gene in the first and second cambium rings and in parenchyma cells between the first and second cambium rings of transgenic sugar beet taproot was significantly higher than that of WT (**Figure 6F**). The expression of the *BvXTH33* gene in the parenchyma cells between the first and second cambium rings and in the first and third rings of transgenic taproots was significantly higher than that of the WT, but there was no consistent significant difference in the expression of the *BvFAD3* gene in each cambial ring of transgenic plant taproots compared with the WT (**Figures 6G, H**). The BvCEL activity in each cambial ring and in parenchyma cells between the second and third rings of transgenic sugar beet taproots was significantly lower than that of the WT (**Figure 7E**). The BvCESA activity in parenchyma cells between each cambial ring of transgenic sugar beet taproots was significantly higher than that of the WT (**Figure 7F**). Compared with the WT, transgenic plant taproots exhibited higher BvFAD activity in the first cambium rings and in parenchyma cells between the first and second cambial rings (**Figure 7G**). The BvXTH activity in parenchyma cells between each cambial ring and in the first cambial ring of transgenic sugar beet taproots was significantly higher than that of the WT (**Figure 7H**). These results suggest that BvBZR1 can be involved in regulating the expression of genes related to cell wall synthesis in each cambium ring and parenchyma cell of sugar beet taproots, improving its enzyme activity, thereby promoting the growth of parenchyma cells and the development of each cambium ring, leading to lateral thickening of the taproot.

### *BvBZR1* regulated enzyme activity related to sucrose metabolism, thereby promoting sucrose accumulation in transgenic sugar beet taproots

**Figure 8:**
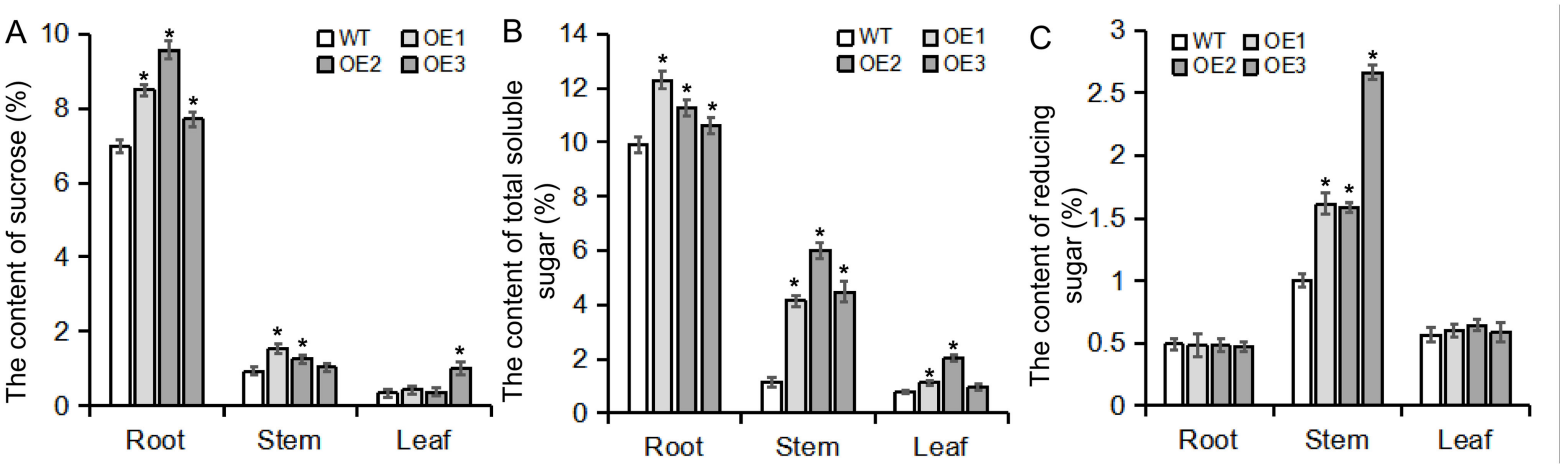
Sugar content in the roots, stems, and leaves of transgenic plants. The contents of sucrose **(A)**, total soluble sugar **(B)**, and reducing sugar **(C)** were measured in the roots, stems, and leaves of transgenic plants. Data are expressed as means ± standard errors (n = 3). An asterisk (*) indicates a significant difference (Duncan’s multiple range test: WT vs. transgenic plants, P < 0.05).

Previous transcriptional KEGG enrichment analysis revealed that the DEGs were significantly enriched in starch and sucrose pathways; therefore, the contents of sucrose, soluble sugars, and reducing sugars were detected in the roots, stems, and leaves of transgenic plants (**Figures 8A–C**). In taproots, the accumulation of sucrose and total soluble sugars in transgenic plants was significantly higher than that in WT plants, while there was no significant difference in the accumulation of reducing sugars. In the stems, transgenic plants exhibited higher contents of reducing sugars and total soluble sugars compared with the WT. In the leaves, there was no consistent significant difference in the contents of sucrose, reducing sugar, and soluble total sugar in the leaves (**Figures 8A–C**). These results indicate that *BvBZR1* can regulate sucrose accumulation in the taproot.

**Figure 9:**
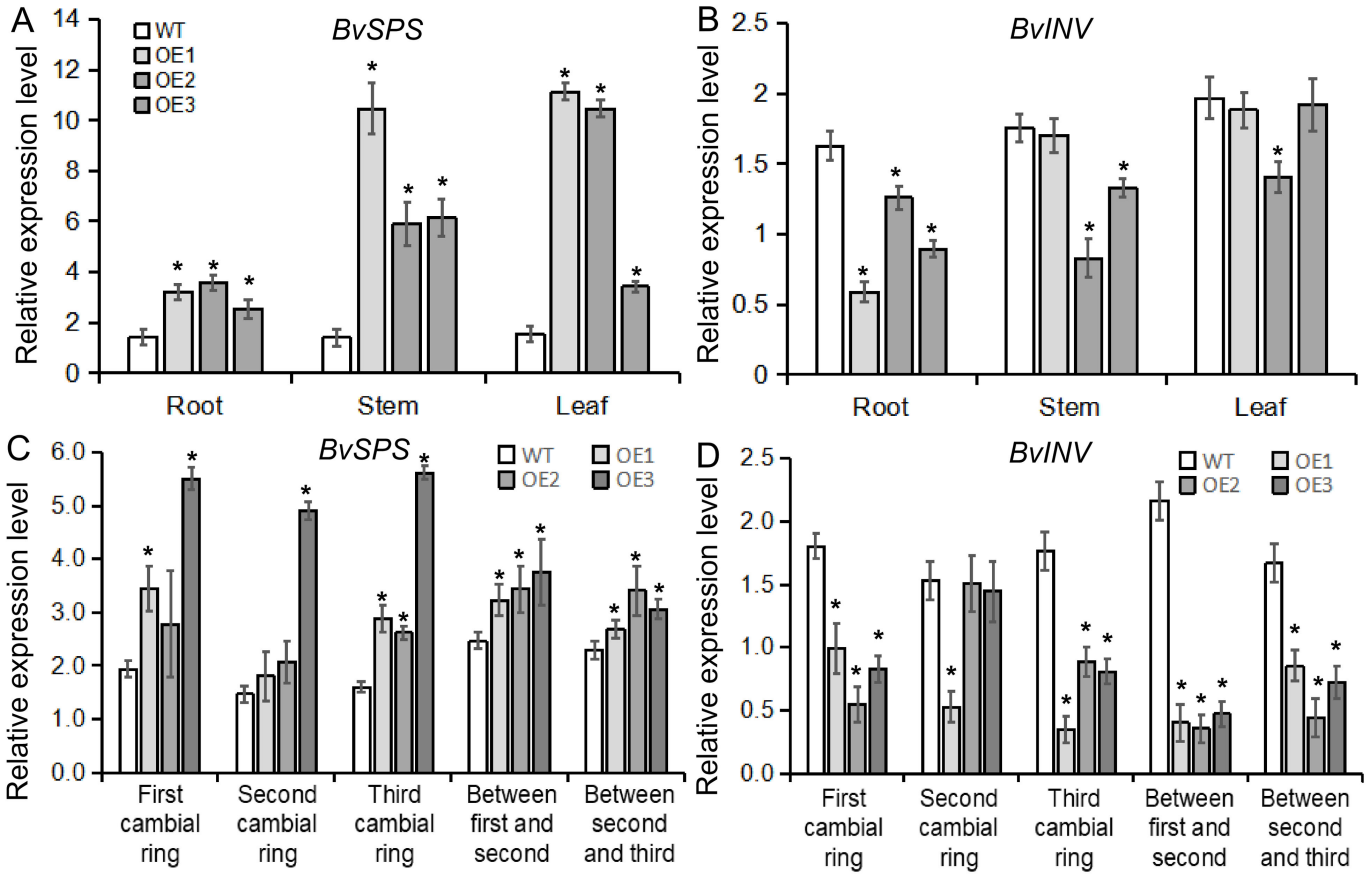
The expression patterns of genes related to sucrose metabolism in BvBZR1 transgenic sugar beet. The expression level of *BvSPS* **(A)** and *BvINV* **(B)** in the roots, stems, and leaves of transgenic plants, and the expression level of *BvSPS* **(C)** and *BvINV* **(D)** in the cambial rings of transgenic plant taproots. Data are expressed as means ± standard errors (n = 3). An asterisk (*) indicates a significant difference (Duncan’s multiple range test: WT vs. transgenic plants, P < 0.05).

**Figure 10:**
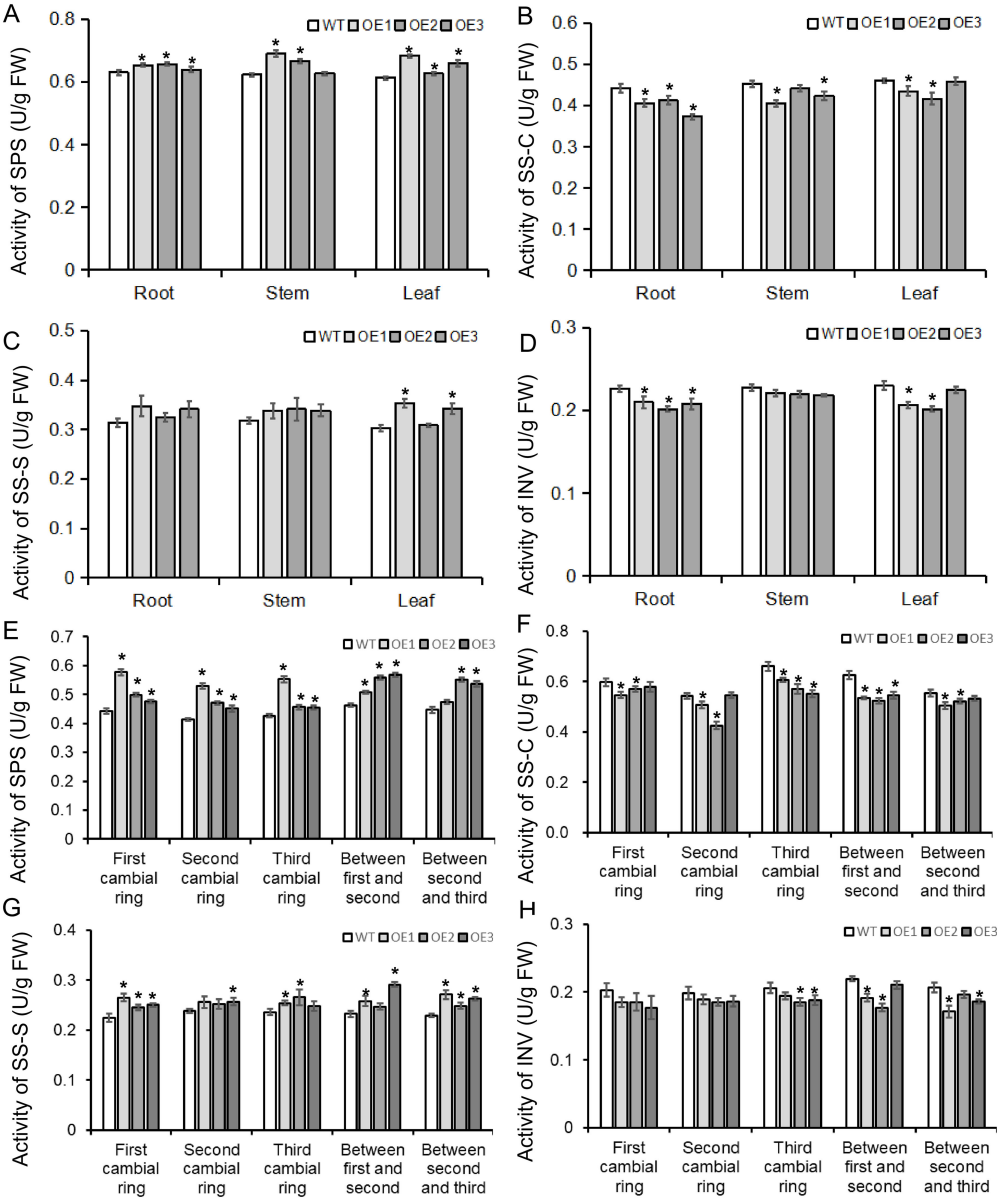
The activity of enzymes related to sucrose metabolism in BvBZR1 transgenic sugar beet. The activity of BvSPS **(A)**, BvSS-C **(B)**, BvSS-S **(C)**, and BINV **(D)** in roots, stems, and leaves of transgenic plants, and the activity of BvSPS **(E)**, BvSS-C **(F)**, BvSS-S **(G)**, and BINV **(H)** in cambial rings of transgenic plant taproots. Data are expressed as means ± standard errors (n = 3). An asterisk (*) indicates a significant difference (Duncan’s multiple range test: WT vs. transgenic plants, P < 0.05).

To better understand how the *BvBZR1* gene regulates sucrose accumulation in sugar beets, gene expression and enzyme activity related to sucrose metabolism were detected in transgenic plants. *BvSPS* expression in transgenic sugar beet taproots, stems, and leaves was significantly higher than that of the WT, while *BvINV* expression in transgenic plant taproots was significantly lower than that of the WT (**Figures 9A, B**). The activity of enzymes related to sucrose metabolism showed that in the taproot, BvSPS activity was significantly higher in transgenic plants than in the WT. The activities of BvSS-C and BvINV were significantly lower than those in the WT, while BvSS-S activity showed no significant changes. However, in stems and leaves, there was no consistent significant difference between transgenic and WT plants (**Figures 10A–D**). These results indicate that *BvBZR1* can regulate the expression levels of the *BvSPS* and *BvINV* genes, increase sucrose phosphate synthase activity, and reduce sucrose synthase (decomposition direction) and sucrose invertase activity, thereby increasing sucrose accumulation in taproots.

In addition, the expression levels of genes and the activities of enzymes related to sucrose metabolism were detected in each cambial ring and in inter-ring parenchyma cells of transgenic plant taproots. *BvSPS* expression in the parenchyma cells between the second and third rings and in the third cambium rings of transgenic taproots was significantly higher than that of the WT. *BvINV* expression in each inter-ring parenchyma cell and in the third cambium ring of transgenic taproots was significantly lower than that of the WT (**Figures 9C, D**). BvSPS activity was significantly higher in the parenchyma cells between the first and second rings and in each cambium ring of transgenic taproots than in the WT. BvSS-C activity was significantly lower in each inter-ring parenchyma cell and in the third cambium ring of transgenic taproots than in the WT. BvSS-S activity was significantly higher in the parenchyma cells between the second and third rings and in the first cambium rings of the transgenic taproot than in the WT (**Figures 10E–H**). These results indicate that *BvBZR1* can increase the activity of sucrose phosphate synthase in the cambium ring and sucrose synthase (synthesis direction) in the inter-ring parenchyma cells and reduce the activity of sucrose synthase (decomposition direction) in the cambium ring and inter-ring parenchyma cells, thus increasing the sucrose content in the taproot.

## Discussion

Brassinosteroids (BRs) are natural plant hormones that are critical for root growth and development. For example, a low exogenous BR concentration significantly stimulated root elongation in Arabidopsis, tomato, and wheat (Roddick et al., 1993; Müssig et al., 2003; Avalbaev et al., 2021). Mutants defective in BR biosynthesis, such as *dwf1-6*, *det2*, and *cpd*, have much shorter roots than those of WT plants, which can be rescued by exogenous application of BRs (Chory et al., 1991; Fujioka et al., 1997; Müssig et al., 2003). Our previous research results showed that 0.1 mg/L BR treatment of sugar beet seedlings for 10 days promoted the development of parenchyma cells and the secondary xylem and significantly increased taproot diameter (Wang et al., 2021). *BvCPD* overexpression significantly increased the endogenous BR content, promoted the development of parenchyma cells and vascular bundles, and increased the taproot diameter, while the RNAi lines inhibited the growth and development of sugar beet taproots (Guo et al., 2023). BZR1, as an important transcription factor in the BR signal transduction pathway, plays an important role in regulating plant root meristem size, root cell elongation, and root hair development (Chaiwanon and Wang, 2015; Wei and Li, 2016; Nolan et al., 2023). In the present study, *BvBZR1* was significantly induced by BR expression, and transgenic sugar beet overexpressing *BvBZR1* exhibited a higher root length and weight and larger taproot diameter compared with the WT due to enhanced spacing between cambial rings, which increased the size and layers of parenchyma cells, indicating that *BvBZR1* can improve the growth and development of sugar beet taproots.

Recent studies have shown that BR can promote the expression of genes related to cell wall metabolism, improve cell wall biosynthesis, and thereby promote plant root cell elongation. For example, the relative expression level of *BRI1* in neighboring epidermal cells determines root cell elongation. Ectopic expression of *BRI1* in nonhair cells inhibits root cell elongation, which may occur because high BRI1 activity in nonhair cells upregulates the expression of *ACS* genes, resulting in an increase in ethylene and accumulation of crystalline cellulose in the cell wall of nonhair cells, impairing unidirectional cell elongation, and inhibiting overall root elongation (Fridman et al., 2014). Consistently, expression of *bzr1-1*D (an activated and hypophosphorylated form of BZR1) in the epidermis can rescue the cell length within the elongation zone and partially rescue the final root length of *bri1-116* (Chaiwanon and Wang, 2015). *BvBZR1* overexpression increased the areas of parenchymal cells between cambial rings, indicating that *BvBZR1* can promote the growth and development of parenchymal cells in sugar beet taproots. In sugar beet, external BR application can significantly increase the areas and layers of parenchymal cells between cambial rings and enhance secondary xylem development. BR can promote cell wall growth by regulating genes associated with cell wall biosynthesis, such as *BvXTH33*, *BvCESA6*, *BvSHV3*, *BvPARVUS*, and *BvCEL1* (Wang et al., 2021). *BvCPD* significantly increases the accumulation of lignin and cellulose, the main components of the cell wall, between cambial rings, which provides a reliable basis for the development of vascular bundles (Guo et al., 2023). Similarly, the BR biosynthetic mutant *cpd*, the BR receptor mutant *bri1*, and the BR signaling mutant *bin2* exhibit negative cell wall metabolism, while overexpression of the *BRI1* and BR signaling genes in plants shows positive cell wall biosynthesis, resulting in more vascular bundles (Choe et al., 1999; Ibañes et al., 2009). In the present work, the transcriptome showed that the *BvBZR1* may be involved in regulating plant cell wall tissue metabolism and the xyloglucan metabolic process, and yeast single hybridization and dual-LUC assays further verified that *BvBZR1* could directly bind the promoter region of the *BvXTH33* and *BvCESA6* genes. Transgenic sugar beet exhibited a higher expression level of *BvXTH33, BvCESA6*, and *BvFAD3*, but a lower expression level of *BvCEL1* in each cambial ring and in parenchyma cells between each cambium ring. The activity of BvCEL, BvCESA, BvFAD, and BvXTH enzymes exhibited similar regulatory trends, indicating that *BvBZR1* can be involved in regulating the expression of genes related to cell wall synthesis in each cambium ring and parenchyma cell of the sugar beet taproot, improving its enzyme activity and thereby promoting the growth of parenchyma cells and the development of each cambium ring.

Recent studies have shown that BR signaling may play an important role in regulating plant sugar metabolism. For example, the downregulation of BR receptor kinase BAK1 (BRI1-associated kinase 1) expression leads to a decrease in soluble sugar levels in sugarcane (Vicentini et al., 2009). The BR biosynthesis-deficient mutant *cpd* and the *cbb1* mutant have lower sugar accumulation; similarly, exogenous BRz application results in a significant decrease in sugar accumulation in *Arabidopsis* (Schroder et al., 2014). The sterol reductase DIMINUTO1 mutant dx exhibits a lower level of endogenous BR in tomato, ultimately leading to a decrease in soluble sugars and starch in the fruit (Lisso et al., 2006). The functional deficient mutants of *BRI1* and *BAK1* exhibit insensitivity to sucrose treatment, which may be because the interaction *in vivo* and *in vitro* between BRI1 and BAK1 with a G protein is regulated by the balance of sucrose homeostasis (Peng et al., 2018). In addition, sucrose can positively regulate the transcription of the brassinosteroid-activated transcription factor *BZR1* gene and stabilize the BZR1 protein (Zhang et al., 2015), and the BZR1 protein can act on the glucose sensor HXK1 to regulate glucose metabolism (Zhang and He, 2015). BZR1 can interact with the alkaline leucine zipper transcription factor GBF2, enhancing β-amylase activity and degrading starch in stomatal guard cells (Reinhold et al., 2011; Li et al., 2020). The BR signaling factor BSK positively regulates BR signal transduction by upregulating the transcription and protein levels of *BSU1*; BSK8 interacts with BSL to dephosphorylate sucrose phosphate synthase (SPS), activating SPS and promoting sucrose metabolism in cells (Wu et al., 2014; Ren et al., 2019). Our transcriptome data suggest that *BvBZR1* may be involved in starch and sucrose metabolism pathways. Transgenic sugar beet overexpressing *BvBZR1* exhibits higher contents of sucrose and total soluble sugar in taproots compared with the WT, which may be due to the lower activity of BvINV and BvSS-C and higher activity of BvSPS in the taproots. Further research showed that the higher activities of BvSPS and BvSS-S and lower activities of BvSS-C were found in each cambial ring and in parenchyma cells between each cambium ring of the taproot. The BvINV activity significantly decreased in parenchyma cells between each cambium ring, indicating that *BvBZR1* could regulate the activity of enzymes related to sucrose metabolism, maintain relatively high sucrose and soluble sugar contents in the taproots, ensure relatively active sugar metabolism, and promote the growth and development of sugar beet taproots.

## Conclusion

In summary, *BvBZR1* overexpression significantly increased the taproot diameter due to a significant enhancement in the spacing between cambial rings by increasing the size and layers of parenchyma cells. *BvBZR1* is involved in regulating the expression of genes related to cell wall metabolism and sucrose metabolism, improving corresponding enzyme activity, accumulating sucrose and soluble sugars to promote the development of each cambium ring and parenchyma cell, and thereby promoting the growth and development of sugar beet taproots.

## Data Availability

The datasets presented in this study can be found in online repositories. The names of the repository/repositories and accession number(s) can be found in the article/**Supplementary Material**.
