# Supplementary material for: BvBZR1 improves parenchyma cell development and sucrose accumulation in sugar beet (Beta vulgaris L.) taproot
Source: Front Plant Sci. 2025 Feb 3;16:1495161. doi: 10.3389/fpls.2025.1495161 (PMC11830749; doi:10.3389/fpls.2025.1495161)
Supplement: Supplementary file 1 [file Table1.doc]

**Table S1** List of the primers used for the experiments.

| BvBZR1-BamHI-F: | 5'-CGGGATCCCGACAAGACACAACCAACGAAGC-3'; |
| --- | --- |
| BvBZR1-SalI-R: | 5'-ACGCGTCGACGTCGGGTCCTACCAGTCCCACTTCC-3' |
| HYG2-F: | 5'-GTTTAGCGAGAGCCTGACCT-3'; |
| HYG2-R: | 5'-GTCGTCCATCACAGTTTGCC-3' |
| BvBZR1-RT-F: | 5'-GCTGGTTGGATTGTTGAGCC-3'; |
| BvBZR1-RT-R: | 5'-GCTTTGTGGAGCAGAAGAAGAG-3' |
| BvActin-F: | 5'-TGCTTGACTCTGGTGATGGT-3'; |
| BvActin-R: | 5'-AGCAAGATCCAAACGGAGAATG-3' |
| BvXTH33-F: | 5'-CCCTGGTTTCACCTCTGGTATT-3'; |
| BvXTH33-R: | 5'-CTCTTCTCTCCCTGTCTTTACACT-3' |
| BvCEL1-F: | 5'-CCGTGATTCCTGGTGTTGCT-3'; |
| BvCEL1-R: | 5'-TGGCACTTGGTGATTGGAGT-3' |
| BvFAD3-F: | 5'-GCTCTATTAGGAATAAACCAGCCC-3'; |
| BvFAD3-R: | 5'-TAAGCGGCGTGCATTTTGTC-3' |
| BvCESA6-F: | 5'-GCCTGTGATGTTTGTGCCTT-3'; |
| BvCESA6-R: | 5'-GGGGGACTACCTTTCTGCTT-3' |
| BvSPS-F: | 5'-CTGCGGCTTATGGATTGCCT-3'; |
| BvSPS-R: | 5'-CCCATTCTGCCTGCACTTTG-3' |
| BvINV-F: | 5'-CCTGTGGGAACTATTGCTGCT-3'; |
| BvINV-R: | 5'-AACCCTTGACCTGGACTGTG-3' |
